# Nanostructure Optimization of Platinum-Based Nanomaterials for Catalytic Applications

**DOI:** 10.3390/nano8110949

**Published:** 2018-11-17

**Authors:** Sibin Duan, Zhe Du, Hongsheng Fan, Rongming Wang

**Affiliations:** 1 Beijing Advanced Innovation Center for Materials Genome Engineering, Beijing Key Laboratory for Magneto-Photoelectrical Composite and Interface Science, School of Mathematics and Physics, University of Science and Technology Beijing, Beijing 100083, China; sibinduan@ustb.edu.cn (S.D.); danglow001@163.com (Z.D.); 2 Department of Physics, Beihang University, Beijing 100191, China; hsfan@buaa.edu.cn

**Keywords:** platinum, nanostructure control, catalytic, structure-property relationship

## Abstract

Platinum-based nanomaterials have attracted much interest for their promising potentials in fields of energy-related and environmental catalysis. Designing and controlling the surface/interface structure of platinum-based nanomaterials at the atomic scale and understanding the structure-property relationship have great significance for optimizing the performances in practical catalytic applications. In this review, the strategies to obtain platinum-based catalysts with fantastic activity and great stability by composition regulation, shape control, three-dimension structure construction, and anchoring onto supports, are presented in detail. Moreover, the structure-property relationship of platinum-based nanomaterials are also exhibited, and a brief outlook are given on the challenges and possible solutions in future development of platinum-based nanomaterials towards catalytic reactions.

## 1. Introduction

Platinum (Pt), with unfilled 5*d* shell electron orbital, possesses rich electronic structure and exhibits excellent catalytic activities in a series of catalytic processes, including automobile exhaust gas treatment, fuel cell, petroleum refining, organic synthesis, and hydrogen production [1,2,3,4,5,6]. The limited Pt storage compels the researchers to reduce the usage of Pt and to promote their catalytic activities by designing specific Pt-based nanomaterials (NMs). Compared with the bulk counterpart, the Pt-based NM has a significantly increased specific surface area and a greatly improved number of exposed active sites, thus, the utilization of Pt atoms is greatly increased [7,8,9]. During the past few years, various wet-chemical methods have been developed for the synthesis of Pt-based NMs. During the wet-chemical synthesis of Pt-based NMs, commonly used Pt precursors are H_2_PtCl_6_, K_2_PtCl_6_, Na_2_PtCl_6_, and Pt(acac)_2_. The solvents include water, ethanol, ethylene glycol, *N*,*N*-Dimethylformamide, oleylamine, and octadecene, while the most common reductants are borohydride, hydrogen, citrate, ascorbic acid, polyol, and amine. Various parameters such as the redox potential, the reaction rate, the polarity and the viscosity of the solvent need to be fully considered for the design of the reaction system [10,11,12]. Pt-based NMs with different nanostructures and dimensions have been extensively investigated, and there are many excellent reviews on this topic [13,14,15,16,17].

**Figure 1:**
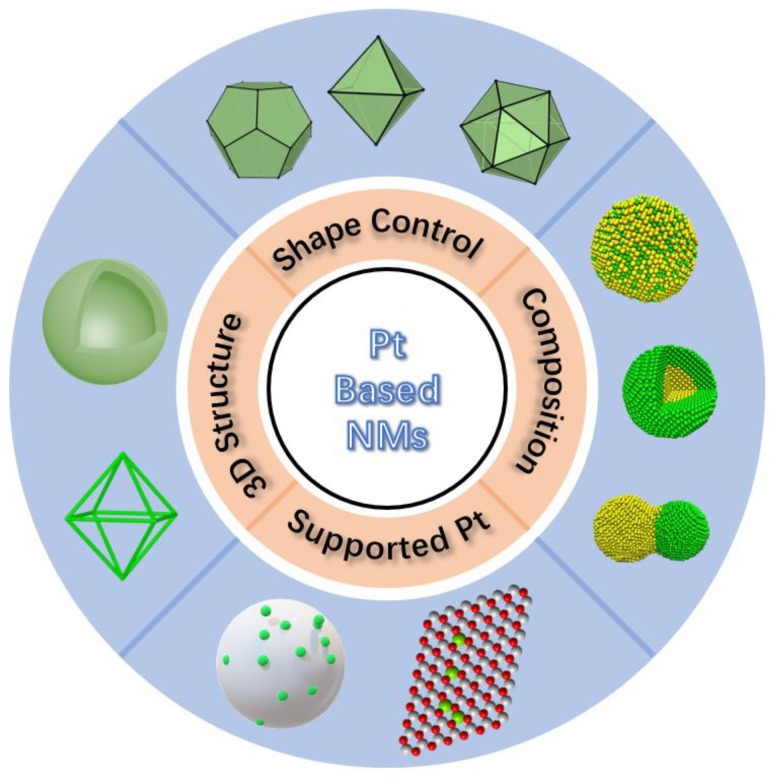
Schematic illustration of four ways of structural regulation of Pt-based NMs.

For Pt-based NMs, thorough characterizing of their nanostructures and catalytic performances in detail with the combination of theoretical simulation and the establishment of structure-property relationship are of both fundamental scientific significance and technological interest [18,19,20]. Various strategies have been developed to optimize their nanostructures, and their catalytic properties are further enhanced accordingly, as shown in Figure 1. (1) The incorporation of another transition metal element, such as Fe, Co, Ni, or their compound, to build a Pt-based alloyed, core-shell or heterogeneous structure, has been developed into a hot spot in the field of Pt-based catalysts research [21,22,23,24,25]. Numerous experimental and theoretical results show that these multi-component structures can not only effectively reduce the usage of precious metal Pt and the cost of catalyst, but also bring superior catalytic features resulting from the synergistic effect and the electronic effect [26,27,28]. (2) The exposed crystal facets of Pt-based catalysts determine the arrangement of surface atoms and the configuration of electrons, which directly affect the adsorption/desorption and decomposition of reactants and intermediate molecules during the catalytic reactions [29]. Controlling exposed crystal facets of Pt-based nanoparticles (NPs) is also a common approach to exploring highly active catalysts [30,31]. A series of Pt-based nanocrystals with regular shapes have been successfully synthesized, including cubes, octahedrons, tetrahedrons, icosahedrons, cubic octahedrons, and concave cubes [32,33]. Among them, nanocrystals with {111} and {100} surfaces exposed are widely studied, and the key parameters to determine their shapes have been highlighted [30,34,35]. The relationship between the crystal plane and the catalytic property provides a good experimental and theoretical foundation for a deep comprehension of the surface catalytic process [36,37,38]. (3) The three-dimensional (3D) nanostructures with great structural stability, such as hollow spheres, nanobowls, hollow nanochains, and nanoframes, are further developed to produce a batch of catalysts with higher active surface areas [2,15,39,40]. In the catalytic tests, these 3D nanostructures not only exhibit higher activities, but also exhibit excellent stability [41]. (4) In recent years, numerous support materials with stable structure, high specific surface area, and abundant anchoring sites have been applied into the synthesis of supported Pt NMs [42,43]. The supported Pt NPs have small size, uniform distribution and considerable active surface area, which are resisted to sintering during the fabrication of NPs and the catalytic reactions [44,45]. More importantly, the metal-support interaction between Pt species and support materials is beneficial to adjust the electronic structure of Pt, thereby further enhances the catalytic performances of the Pt-based NMs [46,47,48].

Herein, we will give an overview of Pt-based NMs with promoted catalytic performances through nanostructure control. Pt-based bimetallic or multimetallic nanostructures synthesized by composition regulation are summarized in Section 2, where the structure-property relationship of alloyed, core-shell, and heterogenous Pt based NMs is proposed. The shape control methods of Pt-based NMs to enhance their catalytic properties are presented in Section 3. In Section 4, the 3D Pt-based NMs with open structure and more accessible surface sites are summarized. We also discuss the metal-support interaction in the supported Pt-based NMs in Section 5, where the Pt single atom catalyst is highlighted. In the last section, we will analyze the scientific problems in the field of Pt-based NMs and give a brief outlook based on the previous summaries.

## 2. Composition Regulation

Pt-based bimetallic or multimetallic nanostructures, due to synergic effect, tend to exhibit superior performances to the single-component Pt [14,16,49]. Pt possesses the best catalytic activity towards molecular alcohol oxidation, because it can efficiently decompose alcohol molecules into CO_x_ or CH_x_ [50,51]. However, the intermediates are incompletely oxidized, which are strongly adsorbed on the surface of Pt atoms, resulting in the catalyst poisoning and reduced catalytic performances [52]. Moreover, Pt does not easily activate H_2_O molecules, so it is not effective to oxidize surface poisoning species [53]. However, although decomposing small organic molecules weakly, some transition metal elements activate H_2_O more easily than Pt, forming OH species adsorbed on the metal atoms [51,54,55]. Thus, it is obvious that the construction of bimetallic nanostructure consists of Pt and another transition metal element, owing to synergistic effect, make it easier to remove CO and OH adsorbed species and eliminate the catalyst poisoning. Density functional theory (DFT) calculations also show that the bifunctional effect of the bimetallic nanostructure does enhance the catalytic activities towards methanol oxidation reaction (MOR) [56]. Due to the changes of neighboring atoms and electronic environments, the surface lattice configuration and electronic structure of Pt atoms are changed accordingly, which impact on the adsorption and dissociation of molecules [57,58]. For instance, the shift of *d*-band center in the transition metal has a fine linear relationship with the variation of the CO species adsorption energy in a certain range during the small organic molecules oxidation reaction [52,53,59]. Optimized design of catalytic properties can be achieved by controllable adjustment of the *d*-band center [44,60,61]. The multi-component system exhibits richer and more tunable physicochemical properties than pure Pt materials. By precisely controlling the thermodynamics and kinetic parameters, it is feasible to fabricate bimetallic or multimetallic nanostructures, such as alloyed, core-shell and heterogeneous nanostructures.

**Figure 2:**
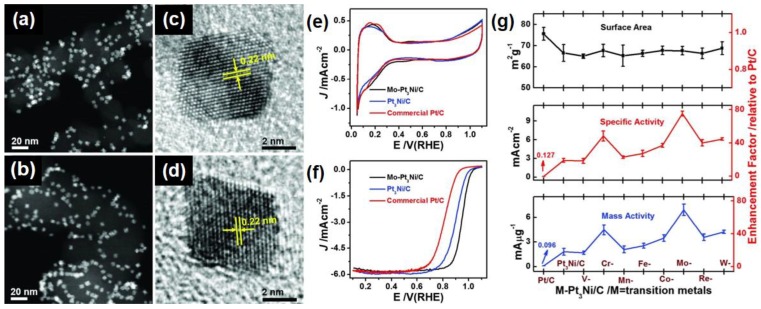
Transition metal doped octahedral Pt_3_Ni/C catalysts: (**a**,**b**) Typical high-angle annular dark field scanning transmission electron microscopy (HAADF-STEM) images of the (**a**) Pt_3_Ni/C and (**b**) Mo-Pt_3_Ni/C catalysts. High-resolution transmission electron microscopy (HRTEM) images of individual octahedral (**c**) Pt_3_Ni/C and (**d**) Mo-Pt_3_Ni/C catalysts. (**e**) Cyclic voltammograms of octahedral Mo-Pt_3_Ni/C, octahedral Pt_3_Ni/C, and commercial Pt/C catalysts recorded at room temperature in N_2_-purged 0.1 M HClO_4_ solution with a sweep rate of 100 mV/s. (**f**) ORR polarization curves recorded at room temperature in an O_2_-saturated 0.1 M HClO_4_ aqueous solution with a sweep rate of 10 mV/s and a rotation rate of 1600 rpm. (**g**) The electrochemically active surface area (top), area specific activity (middle), and mass specific activity (bottom) at 0.9 V versus RHE for these transition metal–doped *M*-Pt_3_Ni/C catalysts. Adapted from [65], with permission from AAAS, 2015.

In order to obtain alloyed NPs, a one-pot synthesis method is generally adopted, that is, controlling the reaction conditions of the mixture of the reactant, the solvent, and the additive, during which different metals are simultaneously reduced [62,63]. Through simply adjusting the molar ratio of Pt and another metal, the binary composition of the resulted alloyed NPs is well controlled. Zhang et al. reported a one-pot strategy to prepare the highly uniform Pd–Pt alloyed NPs with various Pt/Pd atomic ratios supported on graphite nanoplatelets by simply adjusting the molar ratio of the Pd/Pt precursors [64]. As the Pd content increases, the X-ray photoelectron spectrum (XPS) shows that the Pt 4f binding energy gradually decreases, which is due to partial transfer of electrons that is induced by the electronegativity difference between Pt and Pd. The Pd_1_Pt_3_ NPs exhibited the highest activity towards MOR compared with pure Pt NPs and Pd-Pt bimetallic NPs with other atomic ratios, which indicates that a reasonable composition regulation is beneficial to the catalytic effect. Besides the component ratio, the type of additive metal element in a multi-component NP is also proved to be a key factor to modulate the properties of Pt-based NPs. Huang et al. doped Pt_3_Ni octahedrons with *M*, where *M* is vanadium, chromium, manganese, iron, cobalt, molybdenum, tungsten, or rhenium [65]. Among them, molybdenum doped Pt_3_Ni (denoted as Mo-Pt_3_Ni) NPs showed the highest catalytic activity towards oxygen reduction reaction (ORR), and its area and mass specific activity were 81 and 73 times higher than those of commercial Pt/C catalyst, respectively, as shown in Figure 2.

**Figure 3:**
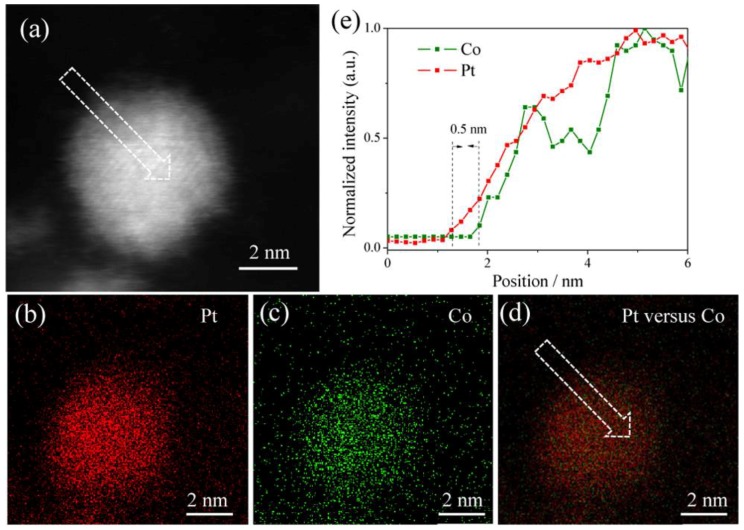
Pt-rich PtCo NPs with Pt-rich shells: (**a**) ADF-STEM image of a Pt_3_Co@Pt NPs. (**b**–**d**) EELS mapping of Pt (red), Co (green), and the composite image of Pt vs. Co. (**e**) Line-profile analysis from the indicated area of (**a**,**d**), demonstrating about 0.5 nm Pt skin thickness. Reproduced from [49], with permission from American Chemical Society, 2016.

For core-shell nanostructure, the surface lattice strain has an important influence on the catalytic properties [26,60]. The Pt-rich surface with moderate lattice compression, which weakens the adsorption strength of oxygen-contained species due to the downward shift of the *d*-band center, exhibits excellent catalytic performances [66,67]. Therefore, fine-tuning the core-shell nanostructure and exploring the appropriate lattice strain have attracted much attention [68,69]. The redox potentials of the metal precursors affect the reduction order and rate of the metal elements [70,71]. The surface energy and stability of the different metal elements in the specific solvent are also different. Taking advantage of these reaction parameters, the component segregated core-shell nanostructure can be obtained. Zhang et al. synthesized Pt-rich PtCo NPs with a wet-impregnation method [49]. Electron energy loss spectroscopy (EELS) suggested that the thickness of Pt-rich shell is about 1–2 atomic layers. It shows superior ethanol oxidation catalytic activity to commercial Pt/C catalysts, as shown in Figure 3. *In situ* Fourier transform infrared spectra and DFT calculations show that Pt_3_Co NPs with Pt-skin promote partial oxidation of ethanol over C–C bond cleavage and are easier to produce acetic acid for ethanol electro-oxidation in contrast to pure Pt. Another common strategy for preparing component segregated nanostructures is dealloyed method, where the more active transition metal is etched from the surface, leaving a relatively stable layer of Pt atoms [60,72]. Through controlling the dealloying extent, the shell thickness and the core composition are well adjusted. For instance, the Pt-Fe NPs can be dealloyed by acid treatment to form a segregated core-shell structure composed of a Pt-rich shell and a PtFe alloyed core [73]. By using the aberration-corrected high-resolution transmission electron microscopy (AC-HRTEM) combined with image simulations, the lattice strain distribution in the NP is directly demonstrated at the atomic scale. The Pt-rich shell is composed of a gradient compressive strain. Due to the lattice strain effect, the catalytic activity of Pt-Fe NPs at 0.9 V (vs. RHE) towards ORR was 1.8 times that of commercial pure Pt catalysts.

**Figure 4:**
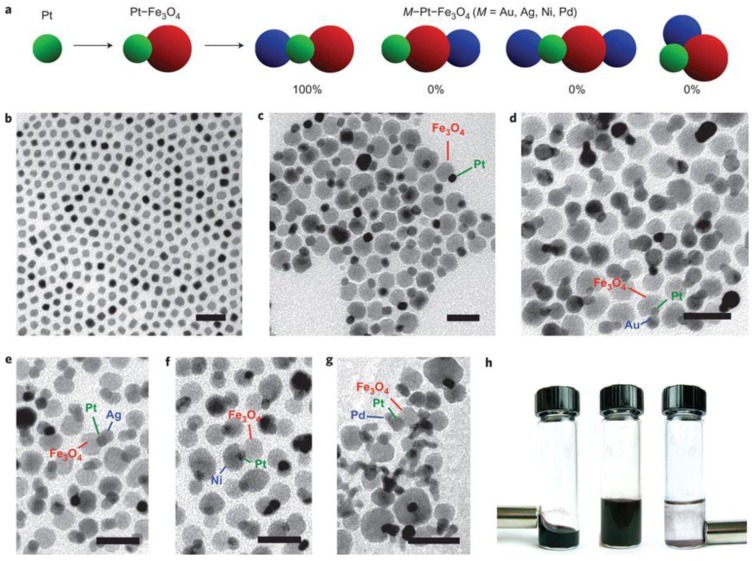
Sequentially seeded growth of *M*–Pt–Fe_3_O_4_ heterogenous nanostructure (*M* = Ag, Au, Ni, Pd). (**a**) Schematic illustration of the chemoselective growth of *M*–Pt–Fe_3_O_4_ heterotrimers, along with the most possible products and their observed frequencies. Representative TEM images of (**b**) Pt NP seeds, (**c**) Pt–Fe_3_O_4_ heterodimers, (**d**) Au–Pt–Fe_3_O_4_, (**e**) Ag–Pt–Fe_3_O_4_, (**f**) Ni–Pt–Fe_3_O_4_, and (**g**) Pd–Pt–Fe_3_O_4_ heterotrimers (scale bar: 25 nm). (**h**) Photographs of a vial containing Au–Pt–Fe_3_O_4_ heterotrimers in hexane (left), which responds to an external Nd–Fe–B magnet, the same vial with Au–Pt–Fe_3_O_4_ heterotrimers in a larger volume of hexanes (middle) and the same vial after precipitation of the heterotrimers with ethanol (right). Reproduced from [28], with permission from Nature Publishing Group, 2011.

The Pt-based multicomponent heterogenous structure is an important research object, where the interface between Pt and another compound directly affects the catalytic activity and selectivity [74,75,76]. Different with the core-shell nanostructure where only Pt atoms in the shells are exposed, the Pt and another component in the heterostructure NPs are all exposed. Most widely used strategy to synthesize the heterostructure NPs is seeded growth method, where one kind of metal atoms are grown on the prepared NPs composed of another component. The interfacial energy between these two kind of metals needs to be well controlled to promote the island growth model rather than the layered growth method [77,78]. Matthew et al. synthesize Pt-based heterostructure NPs using the seeded growth method sequentially [28]. As shown in Figure 4, the Fe_3_O_4_ NP nucleates and grows on a prepared Pt seeds, forming a Pt–Fe_3_O_4_ heterodimer. The Pt–Fe_3_O_4_ heterodimer was further used as a seed to grow *M* (*M* = Au, Ag, Ni, Pd) on its surface, forming a *M*–Pt–Fe_3_O_4_ heterotrimer. The chemoselective growth of the *M*–Pt–Fe_3_O_4_ NPs rather than the Pt–Fe_3_O_4_–*M* NPs is resulted from that the Pt domain serves as an electron sink during the formation of Pt–Fe_3_O_4_ heterodimers, which helps to anchor and reduce the cationic *M* precursors. Sang et al. report a facile one-pot synthesis of CuPt–Cu_2_S, Pt–Cu_2_S heterostructure NPs, and CuPt nanocubes by simply changing the Pt precursor types [75]. Attributing to the synergic effect of Cu and Pt and the interfacial effect between metal and sulfide, these three types of Pt-based NMs shown different catalytic activity and selectivity towards cinnamaldehyde hydrogenation. Moreover, the promoting effect of the heterogeneous structure on the catalytic properties is not only related to the interface species, but also affected by the crystal plane at the interface. Fan et al. used a seeded growth method to prepare Pd nanosheets [76]. The top planes of the Pd nanosheets are covered by (111) facets, while the side planes are dominated by (100) facets. When growing the Pt NPs to the side of the Pd nanosheets, the result NPs were denoted as Pd(100)-Pt interfacial structure. When CTAB was added, the Pt NPs were grown on the (111) facets of the Pd nanosheets, denoted as Pd(111)-Pt. DFT calculations and experimental results show that the difference in binding sites between Pd nanosheets and Pt NPs affects the electron transfer from Pd to Pt atoms, which accordingly affects the catalytic performances. In an alkaline solution, the Pd(100)-Pt interfacial structure exhibited superior catalytic performances to the Pd(111)-Pt interfacial structure and the commercial Pt/C catalyst towards the methanol and ethanol oxidation reactions.

## 3. Shape Control

**Figure 5:**
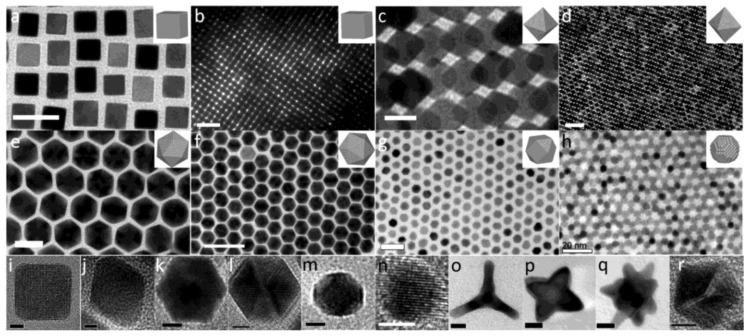
Pt NPs with different shapes: (**a**,**b**,**i**) cubes, (**c**,**d**,**j**) octahedrons, (**e**,**f**,**k**,**l**) icosahedrons, (**g**) cuboctahedrons, (**h**,**n**) spheres, (**m**) truncated cube, (**o**) tetrapod, (**p**) star-like octapod, (**q**) multipod, (**r**) 5-fold twinned decahedron. Scale bars: (**a**,**e**,**g**,**h**) 20 nm, (**b**,**d**,**f**) 50 nm, (**c**,**o**,**p**,**q**) 10 nm, 100 nm, (**i**,**j**) 2 nm, (**k**,**l**,**m**,**n**,**r**) 5 nm. Reproduced from [79], with permission from American Chemical Society, 2013.

The surfaces of NPs tend to expose certain crystal planes to minimize their surface energy. For the face-centered cubic (f.c.c.) crystal structure of Pt-based NMs, the {111}, {100}, and {110} crystal planes have lower surface energy and are preferentially exposed [13,17]. The exposed crystal planes of the NPs determine the arrangement of the surface atoms, which corresponds to the specific surface atomic spacing, the coordination number, and the electronic structure, which have an important influence on their catalytic properties [14,17,29]. For example, the coordination number of the atoms on the Pt(100) and (111) planes is 8 and 9, respectively. The *d*-band center of the (100) planes is closer to the Fermi surface with respect to that of the (111) planes [50]. Controlling the specific exposed crystal planes of Pt NPs not only helps to study the relationship between catalytic activities and nanostructure, but also provides a clear idea for improving the catalytic properties of Pt NPs. The exposed crystal planes of the NPs are directly related to their shape. For the Pt with the f.c.c. crystal structure, the six surfaces of the cubic structure are all (100) facets, while the octahedrons expose the (111) facets. The icosahedral NPs are also exposed with the (111) facets, but it is not single crystalline but with the multi-twinned structure. The common pure Pt polyhedral NPs are shown in Figure 5 [79]. The key to controlling the shape of Pt NPs is to adjust the growth rate of different crystal orientations. Accurate regulation of temperature, solution type, precursors, surfactants, and additives can be applied to adjust the surface energy and growth kinetics of the crystal facets to obtain Pt NPs with various shapes.

The adsorption of carbon monoxide (CO) on the Pt atoms is very strong, and CO is commonly used as a shape controlling agent for Pt NPs growth [80,81]. Wu et al. developed a gas reducing agent in liquid solution method to prepare Pt_3_Ni icosahedral NPs using oleylamine and oleic acid as solvents, CO gas as capping agent and reducing agent to stabilize the {111} facets [80]. Pt-Au and Pt-Pd icosahedrons can also be obtained by changing the precursor and solvent. The area specific activity of the Pt_3_Ni icosahedral NPs was nine times that of the commercial Pt/C catalyst and 1.5 times that of the Pt_3_Ni octahedrons. Because that both the icosahedral and octahedral NPs are all exposed with the (111) planes, the outperformance in catalyzing ORR of the icosahedrons compared with their octahedral counterparts is due to the diffuse elastic strain seen on the surface, induced by stretching the 20 tetrahedrons.

Carbonyls are also commonly used in the shape control of Pt NPs. Kang et al. used Mn_2_(CO)_10_ as shape controlling agent [82,83,84]. Through adjusting the reaction temperature and the Pt(acac)_2_:Mn_2_(CO)_10_ ratio combined with purification of NPs by colloidal recrystallization, monodisperse Pt NPs with the shapes of icosahedrons, truncated cubes, octahedrons, and cubes have been fabricated [79]. It has been found that metal atoms produced by the decomposition of carbonyls may be a key factor in shape control. Using as catalysts in the electrooxidation of formic acid, Pt octahedrons show higher poisoning tolerance than Pt cubes, because that the adsorption of poisoning species is lowest on Pt(111) and highest on Pt(100). Tests on the CO oxidation on Pt NPs with different shapes show that it is structure insensitive in the CO-rich environment, while it is structure sensitive in the O_2_-rich environment. All these catalytic results demonstrate that high quality Pt NPs with different shapes which selectively expose {111} or {100} facets are ideal model catalysts to study structure-property relationship. Additionally, carbonyls can be used to aid the synthesis of nanostructures with high index facets. Wang et al. reported a facile synthesis route to preparing high quality Pt_3_Co nanocubes with a concave structure terminated with high-index crystal facets [84]. Tungsten decomposed from W(CO)_6_ play a partial role in the formation of concave Pt-based NPs.

Various organic agents, with different binding capacities with different crystal facets, have been widely used in the shape control of Pt-based polyhedral NPs [23,32,79]. Through controlling the kind and usage of organic surfactant, polyhedral Pt-based NMs with different exposed facets have been synthesized. Kang et al. used oleylamine and oleic acid as stabilizers and reducing agents, Pt(acac)_2_ and Zn(acac)_2_ as precursors to obtain PtZn spherical alloyed NPs of about 4.5 nm [85]. When benzyl ether was added as a surfactant, PtZn alloyed cubes of about 6.9 nm were obtained. The MOR activity on spherical PtZn NPs is higher than that on cubic PtZn NPs because of less carbonaceous species accumulation. Bu et al. adopted cetyltrimethylammonium chloride (CTAC) as shape controlling agent in the oleylamine system to obtain Pt_3_Co hierarchical zig-zag structures terminated with high index crystal planes [86]. The specific activities towards ORR of the Pt_3_Co hierarchical zig-zag structures were 39.6 times that of the commercial Pt/C catalyst. DFT calculations reveal that the active three-fold hollow sites on the Pt-rich high-index facets are key in promoting ORR activities.

Many inorganic ions also have a shape controlling effect on the formation of Pt-based NPs [87,88]. Yin et al. used hydrothermal method and C_2_O_4_^2−^ as the (111)-facet selective agent to form Pt-Pd tetrahedrons with an average size of 4.9 nm [89]. The exposed four facets were all (111) planes. While a large amount of Br^−^ and a small amount of I^−^ were added at the same time, Pt-Pd cubes with an average size of 8.5 nm were formed, and the (100) planes were exposed. The MOR test in a perchloric acid solution showed that the cubic NPs exhibited higher activity, while the tetrahedral NPs possessed better cyclic stability, indicating different catalytic properties of the (111) and (100) crystal planes.

## 4. Construction of 3D Structures

The practical catalytic reactions are usually conducted in the relatively harsh environment. The NPs, especially the clusters with small size, are prone to aggregating and sintering into bigger particles, resulting in the loss of the active surface area, and the catalytic performances decline accordingly [90,91,92]. Constructing 3D nanostructures can effectively solve the sintering problem, so that the structure and catalytic performances keep stable during the reactions [15,93,94,95]. Up to now, Pt-based 3D NMs with different shapes, including hollow structure, nanocages, and nanoframes, have been successfully synthesized [39,40,95,96,97,98]. In general, taking advantage of the sacrificial template, galvanic replacement, and Kirkendall effect, the corresponding hollow structure and nanocages can theoretically be obtained. Further acid etching or electrochemical treatment will induce the formation of framework structure.

**Figure 6:**
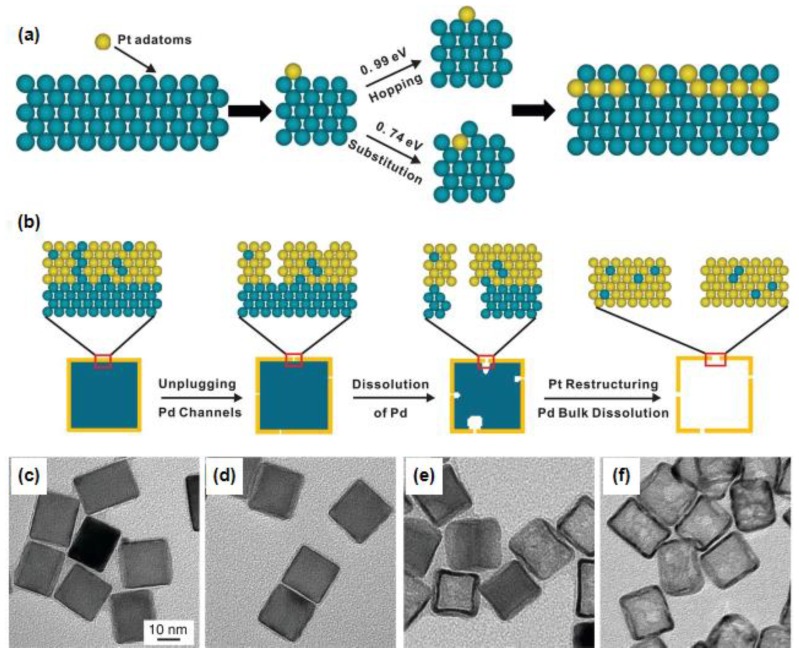
Scheme illustration of Pt-based nanocages with subnanometer-thick walls: (**a**) Pt atoms deposited on the Pd surface may diffuse (“hop”) across the surface or substitute into the surface, leading to a mixed outer-layer composition. (**b**) Schematic of the major steps involved in the continuous dissolution of Pd atoms from a Pd@Pt_4L_ cube to generate a Pt cubic nanocage. (**c**–**f**) TEM images of Pd@Pt_4L_ cubes after Pd etching for (**c**) 0, (**d**) 10, (**e**) 30, and (**f**) 180 min. Adapted from [96], with permission from AAAS, 2015.

In general, it is quite difficult to prepare a single-crystal hollow structure with a specific geometric shape. Xia’s group proposed a feasible solution in this field [69,96,99]. As shown in Figure 6, Pt-Pd core-shell NPs of the cubic or octahedral morphology were firstly fabricated by depositing a few layers of Pt atoms as conformal shells on Pd nanocrystals with well-defined facets. Secondly, the hollow Pt NMs were synthesized by etching away the Pd templates. During this process, the etching is initiated via a mechanism that involves the formation of vacancies through the removal of Pd atoms incorporated into the outermost layer during the Pt deposition. The hollow Pt NMs enclosed by different facets exhibit distinctive catalytic performance towards ORR, demonstrating the structural dependent catalytic properties. DFT calculations reveal that the nanocage models showed substantially enhanced activity relative to their core-shell counterparts attributing to the shortening of Pt-Pt interatomic distances.

**Figure 7:**
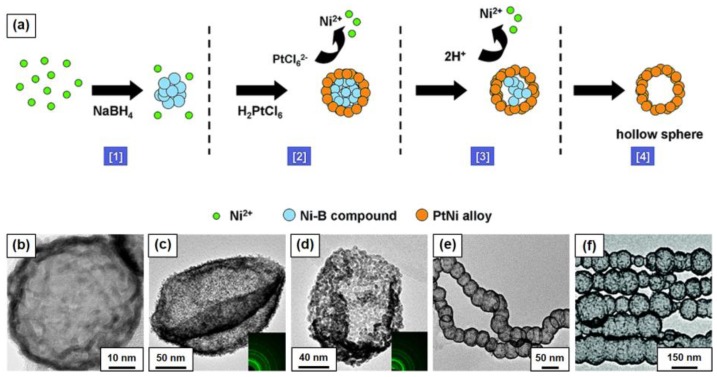
Hollow Pt based NMs with the combination of sacrificial template method and galvanic replacement reaction: (**a**) Schematic illustration for the formation of NiPt hollow spheres, adapted from [105], with the permission from the Royal Society of Chemistry, 2015. TEM images of (**b**) NiPt hollow sphere, adapted from [103], with the permission from the Royal Society of Chemistry, 2011, (**c**) NiPt double-layered nanobowls, adapted from [106], with the permission from Tsinghua University Press and Springer-Verlag BerlinHeidelberg, 2016, (**d**) NiPt single-layered nanobowls, adapted from [106], with the permission from Tsinghua University Press and Springer-Verlag BerlinHeidelberg, 2016, (**e**) CoPt hollow nanochains, adapted from [104], with the permission from American Chemical Society, 2012, and (**f**) FePt oriented hollow nanochains, adapted from [107], with the permission from the Royal Society of Chemistry, 2016.

The synthesis route with the combination of sacrificial template method and galvanic replacement reaction has been shown effective for obtaining hollow Pt-based NPs. In this method, the morphology of prefabricated sacrificial template determines the final morphology of the Pt-based hollow NM to some extent, thus the sacrificial template with regular shapes can be used to prepare the corresponding hollow structure [97,100]. In this fields, Wang’s group conducted systematic work, as shown in Figure 7. Firstly, the *M* (*M* = Ni, Co, or Fe) precursor is reduced by NaBH_4_ with polyvinylpyrrolidone as surfactant at room temperature, forming *M*-based solid spheres. Secondly, Pt precursor is added to the solution containing *M*-based solid spheres. After the galvanic replacement between *M*-based solid spheres and Pt precursor, Pt-Ni hollow spheres, Pt-Ni layer controlled nanobowls, Pt-Co and Pt-Fe hollow nanochains can be obtained [101,102,103,104,105,106,107,108,109,110]. All these Pt-bases hollow NMs show excellent catalytic properties towards CO oxidation, MOR or ethylene glycol oxidation. Dubau et al. used a similar method to synthesize porous hollow PtNi/C nanocatalysts with the help of Kirkendall effect [111]. The composition and crystal structure of the NPs were studied in detail, and the characteristics of Pt-rich surface and lattice compression were proposed. Due to their increased surface area to volume ratio and molecular accessibility and the weakened oxygen binding energy induced by the contracted Pt lattice parameter, the mass and area specific activity of the hollow PtNi/C towards ORR was four and three-fold that of the solid PtNi NPs catalyst, respectively, which highlight the importance of nanoporosity on the catalytic enhancement.

**Figure 8:**
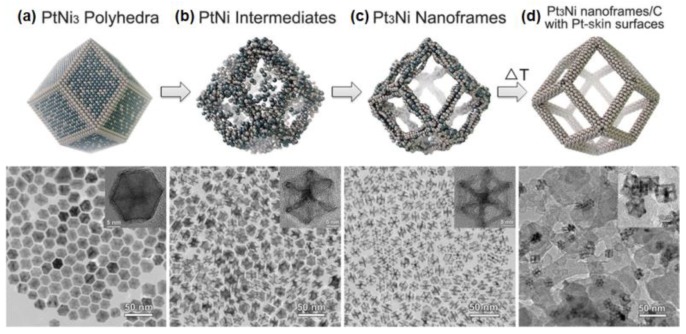
Schematic illustration of the evolution process from polyhedrons to nanoframes: (**a**) initial solid PtNi_3_ polyhedrons, (**b**) PtNi intermediates, (**c**) final hollow Pt_3_Ni nanoframes, and (**d**) annealed Pt_3_Ni nanoframes with Pt(111)-skin–like surfaces dispersed on high–surface area carbon. Reproduced from [115], with permission from AAAS, 2014.

The nanoframes, possessing open structure and more accessible surfaces, have received the researchers’ attention in recent years. Many literatures have proven than the fabrication of nanoframes is a better choice to construct excellent electrocatalysts [37,98,112,113,114]. Chen et al. firstly synthesized PtNi_3_ polyhedrons in oleylamine that had a rhombic dodecahedron morphology and uniform size distribution [115]. Then in non-polar solvents such as hexane and chloroform, dissolved oxygen will slowly corrode Ni atoms. After two weeks, the initial solid PtNi_3_ polyhedrons are gradually converted into hollow Pt_3_Ni polyhedral nanoframes. The evolution process from polyhedrons to nanoframes is shown in Figure 8. The extraordinarily high activity of the Pt_3_Ni nanoframes towards ORR is closely related to the formation of the Pt_3_Ni–Pt-skin with a topmost Pt-skin thickness of at least two monolayers rather than one monolayer, which is common for ideal bulk Pt_3_Ni(111) single crystal. DFT calculations also confirm that Pt_3_Ni(111) with Pt-skin thickness of 2–3 monolayers is optimal for ORR. The proposed synthesis method of nanoframes was also applied to other Pt-based electrocatalysts such as PtCo, PtCu, Pt/Rh-Ni, and Pt/Pd-Ni [95,113,114]. Wu et al. fabricated truncated octahedral PtNi_3_ NPs using solvothermal method, of which were then deposited with a small amount of Au atoms on the angles under the displacement reaction with HAuCl_4_ [116]. Afterwards, the oxidation corrosion of surface Ni atoms was proceeded with the addition of dimethylglyoxime, while Pt and Au atoms were retained to form trimetallic hybrid Au islands on the Pt_3_Ni nanoframes. This ternary composition has an optimized electronic structure and exhibits superior catalytic activity towards 4-nitrobenzaldehyde hydrogenation and MOR to the Pt_3_Ni truncated octahedron and Pt_3_Ni truncated octahedral nanoframes.

## 5. Supported Pt-Based NMs

**Figure 9:**
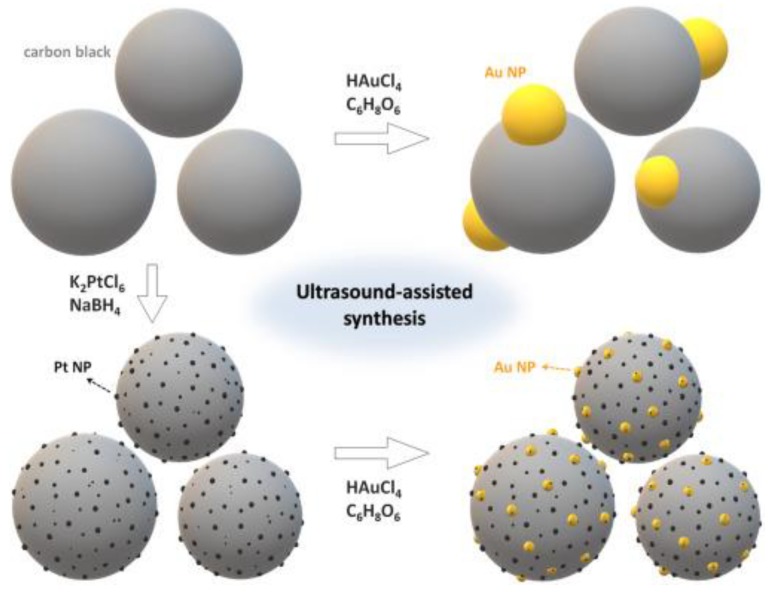
Schematic illustrations of the synthesis procedures of Pt/C, Pt-Au/C and Au/C structures. Reproduced from [121], with permission from Elsevier, 2018.

The nano-sized Pt-based particles face serious agglomeration problem, which make their catalytic performances decreased. In order to maintain good dispersibility and stability of the Pt-based NPs during catalytic processes, it is necessary to deposit the Pt NPs onto the support materials [117,118,119]. There are several basic requirements for the support materials, including a high specific surface area, abundant Pt anchoring sites, the high stability, and good dispersion. For the electrocatalytic application, the support material is also required to have great electrical conductivity. The commonly used supports are carbon-based materials, because they not only meet the above requirements, but also are inexpensive [38,44,74,120]. Recently, we designed and synthesized Pt-Au/C catalysts for catalyzing the formic acid electrooxidation (FAO) reaction, as shown in Figure 9 [121]. During the synthesis, ultrasound-assisted method enabled the uniform and dense loading of Pt and Au on carbon black without any surfactant. The Pt-Au/C catalyst with a Pt:Au atomic ratio of 32:68 exhibited high FAO catalytic activity, which is 153 times higher than that of Pt/C. The extraordinary catalytic performance is attributed to the optimized synergistic effect of Pt and Au.

Graphene, as one kind of two-dimensional carbon material, is stable and has an ultrahigh specific surface area, attracting much attention in recent years [122,123,124]. Shen et al. prepare a multilayer graphene nanosheet (GNS) with a specific surface area of about 247 m^2^/g using a chemical vapor deposition method [124]. Then Pt/GNS catalyst was synthesized using the polyol-assisted reduction method. By simply changing the concentration of H_2_PtCl_6_, Pt/GNS with different Pt loadings can be obtained. The size of Pt NPs is varied from 1.4 nm to 2.1 nm with the increase of Pt loading. XPS analysis showed that the Pt 4f binding energy gradually increased as the NP size decreased. Using as the MOR catalyst, the Pt/GNS with the smallest Pt NP size showed the highest catalytic activity and best stability, which could be resulted from abundant low-coordination atoms at the edges and corners in the sub-nano- or nano-clusters.

Metal carbides can also be used to support Pt NPs [18,125,126]. Qiu et al. reported a urea-assisted ethylene glycol reduction method to form Pt NPs with the size of 3 nm anchored onto the bark-structured TiC nanowires [126]. The Pt/TiC catalyst exhibited improved electrocatalytic activity and good long-term durability toward MOR, in comparison with the commercial Pt/C catalyst. The enhancement of its catalytic performance is mainly attributed to good electron conductivity, large specific surface area, fast transport and short diffusion paths for the reaction, and uniform dispersion of Pt NPs. Jackson et al. loaded Pt NPs onto boron carbide, which exhibited 50–100% activity enhancement and better cyclic stability towards ORR under acidic condition relative to commercial Pt/C catalysts [18]. XPS analysis showed that the boron carbide support has a significant electronic regulation effect on the supported Pt NPs, demonstrating that the electron interaction between the metal and the support has an important regulation effect on the catalytic performance.

**Figure 10:**
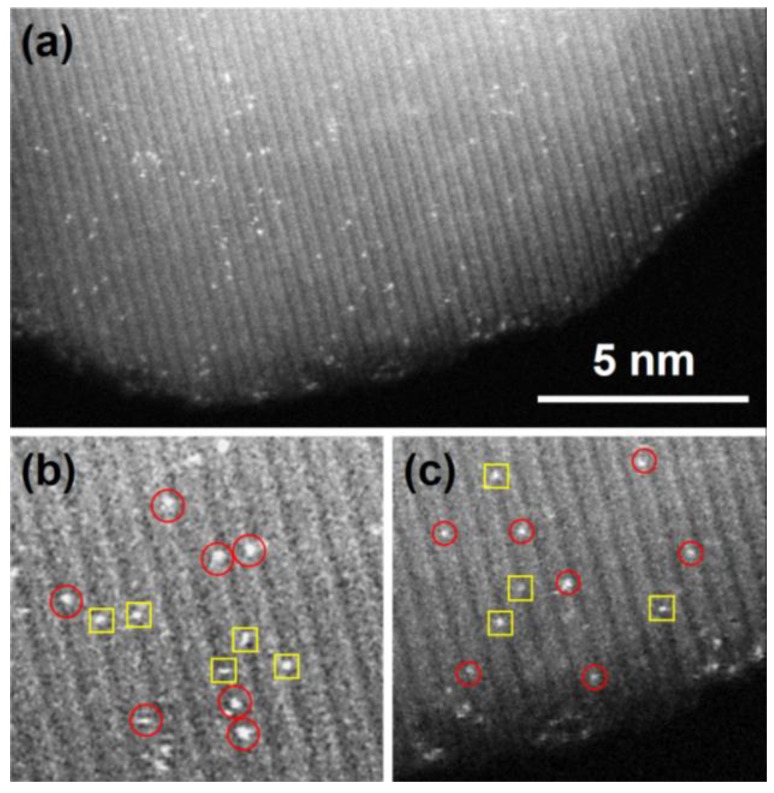
Pt_1_/Fe_2_O_3_ supported single atom catalyst. (**b**) and (**c**) are the enlarged HAADF-STEM images of two parts in image (**a**) for Pt_1_/Fe_2_O_3_ SAC. The red circles indicate the isolated Pt atoms occupied Fe-top positions, and the yellow squares indicate that the isolated Pt atoms possess O-top positions. Reproduced from [133], with permission from IOP Publishing, 2018.

The size effect plays a critical role in determining the metal-support interaction, which is key to affect the catalytic activity and selectivity for a supported Pt catalyst [127]. The reduction of Pt size is limited to a single atom. Single-atom catalysts (SACs), where the isolated individual Pt atoms anchored onto the support surfaces, provide the effective utilization of Pt atoms and open a new research field of catalysis [45,46,128,129,130,131,132]. John et al. obtained Pt single atoms supported on CeO_2_ via a novel atom trapping method [130]. When CeO_2_ powders were mixed with a La-Pt/Al_2_O_3_ catalyst and aged in air at high temperature, the Pt atoms in the La-Pt/Al_2_O_3_ are vaporized in the form of PtO_2_, and are captured by CeO_2_ in a highly dispersed form due to their strong interaction with the CeO_2_ surfaces. In addition, the ability to stabilize Pt atoms of CeO_2_ with different shapes is also different due to the difference in the metal-support interaction between different exposed crystal planes. Accordingly, their catalytic properties towards CO oxidation is also promoted by fine regulation of Pt-CeO_2_ metal-support interaction. Duan et al. reported a modified adsorption method to anchor Pt atoms dispersedly on the Fe_2_O_3_ surfaces and then investigated the mobility of these supported Pt single atoms under various gas environments that are relevant to CO oxidation, water–gas shift, and hydrogenation reactions, as shown in Figure 10 [133]. It is verified that Pt single atoms are stable under the oxidative gas environment, while the presence of either CO or H_2_ molecules in the gas environment facilitates the movement of the Pt atoms and the addition of H_2_O molecules to the CO or H_2_ significantly accelerates the sintering process of the Fe_2_O_3_ supported Pt single atoms.

## 6. Summary and Outlook

The area of Pt-based NMs has undergone tremendous expansion over the past few years. As discussed in this review, Pt-based NMs with tunable nanostructures show great potential in the field of the energy-related and environmental catalysis. Developing various synthesis methods of Pt-based nanocatalysts, controlling their morphologies and compositions, and studying the corresponding catalytic properties are of great significance for exploring active and stable Pt-based catalysts. As reviewed above, through composition regulation, shape control, 3D structure construction and anchoring Pt NPs onto supports, nanostructures and their catalytic properties of Pt-based NMs could be precisely regulated.

Despite the numerous achievements, three problems also exist in the field of Pt-based NMs hindering their applications. (1) The first one is the understanding of structure-property relationship at the atomic scale. The surface/interface structure in the Pt-based NMs determines their catalytic properties. Thus, characterizing the surface/interface structure at the atomic scale is necessary to understand the catalytic mechanism. It can be realized with the development of advanced characterization tools, especially the aberration corrected TEM, which will provide the atomic structure of Pt-based NMs. In addition, the surface/interface structure is changing as the catalytic reaction proceeds, thus, in situ characterization tools need to be developed. Recently, environmental TEM (ETEM) has developed fast and proven to be an efficient way to characterizing the structural evolution under the simulated environment combined with advanced in situ spectroscopy technology. It is believed that aberration corrected ETEM should benefit the deeper comprehend of structure-property relationship in future work. (2) The second one is to further increase the Pt utilization. Even many methods have been developed to enhance the catalytic properties of Pt-based NMs and increase the utilization of expensive Pt, however it is still limited and need further investigation. Lately, the emergence of single-atom catalyst would be the dawn of a real revolution. However, the low-loading and unstable nature of isolated atoms restrict the industrial application of single-atom catalysts. It is supposed that through increasing the surface anchoring sites and modulating the support surfaces could help much to anchoring more stable Pt isolated atoms. (3) The third one is the recycle of Pt-based NMs, which is of great economic benefits. Take the advantage of magnetism, constructing Pt-magnetic hybrid NMs, such as *M*-Pt bimetallic NPs (*M* = Mn, Fe, Co, or Ni) and magnetic oxide supported Pt materials, should be an efficient way to solve this problem.
